# Transiency of postoperative cervical kyphosis seen after surgical correction of sagittal malalignment in adult spinal deformity patients

**DOI:** 10.1371/journal.pone.0254381

**Published:** 2021-07-19

**Authors:** Ki Young Lee, Jung-Hee Lee, Sang Kyu Im, Hae Sung Lim, Gil Han

**Affiliations:** Department of Orthopedic Surgery, Graduate School, College of Medicine, Kyung Hee University, Seoul, Korea; University of California San Francisco, UNITED STATES

## Abstract

**Objective:**

In this study, we evaluated factors affecting changes in cervical lordosis after deformity correction and during follow-up period in adult spinal deformity (ASD) patients with severe sagittal imbalance.

**Methods:**

Seventy-nine patients, with an average age of 71.6 years, who underwent long-segment fixation from T10 to S1 with sacropelvic fixation were included. We performed a comparative analysis of the radiographic parameters after surgery (Post) and at the last follow-up (Last). We calculated the Pearson’s correlation coefficient and performed multilinear regression analysis to predict independent parameters for Post and Last cervical lordosis (CL), T1 slope (T1S), and thoracic kyphosis (TK).

**Results:**

Hyperlordotic changes of -23.3° in CL before surgery was reduced to -7° after surgery, and Last CL had increased to -15.3°. T1S was reduced from 27° before surgery to 14.4° after surgery and had increased to 18.8° at the last follow-up. Through multilinear regression analysis, we found that Post CL and T1S were more significantly affected by the amount of LL correction (p = .045 and .049). The effect of Last T1S was significantly associated with the Last CL; the effect of Last TK, with the Last T1S; and the effect of Post PI-LL, with the Last TK (p < .05).

**Conclusion:**

The postoperative kyphotic change in CL in ASD patients with preoperative cervical hyperlordosis is not permanent and is affected by drastic LL correction and SVA restoration. To achieve spinopelvic harmony proportional to the difference in LL relative to PI, TK becomes modified over time to increase T1S and CL, in an effort to achieve optimal spine curvature.

## Introduction

With increasing life expectancies, adult spinal deformity (ASD) is becoming more prevalent and the demand for surgical treatment of disabilities in the elderly with active lifestyles is also increasing. Maintaining the level of visual gaze and normal upright posture to meet basic human needs, along with sustaining the head-over-pelvis posture, is an important factors for satisfying basic human needs [1–4]. To that end, many studies have already demonstrated the importance of sagittal balance restoration, which involves complex and challenging methods such as long-level constructs and osteotomy in patients with ASD [5–7].

The global sagittal alignment results from a complex chain of correlations. A single segmental or regional change leads to adjacent changes, affecting the shape and position of the whole-spinal curvature [8–11]. When performing long-level fusion, it is important to consider the effects of sagittal alignment and correction of lumbar lordosis on unfused segments. Studies conducted thus far have focused on proximal junctional kyphosis (PJK), sagittal decompensation, pseudarthrosis and pelvic parameters that change after deformity correction of ASD [10–13]. And there is a lack of consensus among studies regarding changes in the cervical spine.

Patients with sagittal malalignment have abnormally increased cervical lordosis (CL) to compensate for the sagittal malalignment and to maintain the horizontal gaze. On the contrary, restoring sagittal balance through deformity correction can lead to problems such as cervical kyphosis [14, 15]. However, studies up to date have examined patients who were relatively young, had varying etiologies, and were treated using various surgical techniques. Furthermore, because the uppermost instrumented vertebras (UIV) in these studies were at relatively high thoracic levels, the studies lacked a clear explanation regarding the correlation of thoracic and cervical reciprocal changes following lumbar lordosis correction.

Therefore, we examined the changes in CL following long-level constructs from T10 to S1 in patients aged 65 years or older with ASD. The patients had drop body syndrome [16] and lumbar degenerative kyphosis (LDK) characterized by pure and severe sagittal malalignment as a single etiology.

## Materials and methods

This study was approved by our Institutional Review Board of Kyung Hee University Hospital (KMC IRB 2017-04-089). The need for informed consent was waived owing to the retrospective nature of the study, and all data were completely anonymized before access.

### Patient selection

This study was a retrospective review of 186 consecutive patients with ASD enrolled from 2008 to 2017.

The inclusion criteria were as follows:

1. Patients aged ≥ 65 years who had ASD accompanied by sagittal malalignment (sagittal vertical axis [SVA] greater than 50 mm, pelvic incidence [PI] minus lumbar lordosis [LL] greater than 10°, and pelvic tilt [PT] greater than 25° with a minimum of 2-year follow-up after deformity correction.
2. Patients who underwent long-segment fixation with sacropelvic fixation, and the UIV was set at the T10 level and the lowermost instrumented vertebra (LIV) at the S1 level as a surgical treatment by a single surgeon at a single institution.
3. Patients who clearly showed atrophy of the back musculature on the cross-sectional area of magnetic resonance imaging and computed tomography scan as a diagnostic criterion for LDK and clinical signs such as walking difficulty with stooping, inability to lift heavy objects to the front, difficulty in climbing slopes, and the need for elbow support when working in the kitchen, resulting in a hard corn on the extensor surface of the elbow [17–19].

The exclusion criteria were as follows: patients with a history of any past cervical or thoracic spinal surgeries, patients with deformities resulting from trauma, spinal infection, ankylosing spondylitis, rheumatoid arthritis, neuromuscular disease, or tumors.

### Radiographic measurements

Sagittal alignment was evaluated using lateral 14×36-inch full spine radiographs obtained by having patients standing in the neutral unsupported position with “fists-on-clavicle” [20]. All digital radiographs were reviewed preoperatively (Pre), 2 months after surgery (Post) and 2 years after surgery (Last), and evaluated using a validated software (Surgimap, Nemaris Inc, New York, NY, USA) [21].

We evaluated PI, sacral slope (SS), PT, thoracic kyphosis (TK), thoracolumbar junction (TL), LL, lumbosacral junction (LS), C7 plumb line (C7SVA), CL, T1 slope (T1S), and C2-7 SVA (C27SVA). Sagittal Cobb angles were measured for CL (C2-7), TK (T5–12), TL (T10–L2), LL (T12–S1) and LS (L4–S1) [22, 23]. Positive values of sagittal cobb angles indicate kyphosis and negative values indicate lordosis.

### Statistical analysis

Statistical analysis was performed using SPSS software (version 20.0; SPSS Inc., Chicago, IL). A paired t-test was used to compare the radiographic parameters after surgery and at the last follow-up. We also calculated the Pearson’s correlation coefficient to analyze the relationship between radiographic parameters including CL and T1S, and multilinear regression analysis of these correlation factors led to predict independent parameters for the Post and Last CL and T1S. The significance level was set to a p-value < .05.

## Results

### Baseline characteristics of the patients

At the time of the study, the database included 186 patients; after applying the inclusion criteria, 79 patients were selected for analysis. The patients consisted of 2 men and 77 women. The mean age at the time of surgery was 71.6 years, and the mean follow-up duration was 57.6 months. Pedicle subtraction osteotomy (PSO) was performed on 64 patients, and oblique lumbar interbody fusion (OLIF) was performed on 12 patients (Table 1).

**Table 1:** Baseline characteristics.

	Patients (N = 79)
**Sex**	
**Female**	77
**Male**	2
**Age at surgery (years)**	71.6 ± 5.4
**Follow-up (months)**	57.6 ± 26.1
**BMI (kg/m**^**2**^**)**	22.4 ± 3.6
**BMD (g/cm**^**2**^**)**	0.968 ± 0.206
**BMD T-score (g/cm**^**2**^**)**	-1.4 ± 1.3
**UIV—T10**	79
**LIV—Sacrum**	79
**Sacropelvic fixation**	79
**PSO**	64
**OLIF**	12

Data are presented as mean ± standard deviation or number.

BMD, bone mineral density; BMI, body mass index; UIV, uppermost instrumented vertebra; LIV, lowermost instrumented vertebra; PSO, pedicle subtraction osteotomy; OLIF, oblique lumbar interbody fusion.

### Radiographic parameters preoperatively, postoperatively, and at last follow-up (Table 2)

**Table 2:** Radiographic parameters preoperatively, postoperatively, and at last follow-up.

Measurement	Pre-operation	Post-operation	Last F/U	p value (Post-Last)
**PI (°)**	56.1 ± 10.8	-	-	-
**SS (°)**	21.4 ± 12.3	45.6 ± 8.9	44.9 ± 8.5	0.382
**PT (°)**	34.6 ± 13.3	12.3 ± 8.2	14.5 ± 10.1	0.032*
**C7SVA (mm)**	188.1 ±78.3	-13 ± 25.1	6.7 ± 27.9	< .001*
**TK (°)**	2.6 ± 13.4	25.7 ± 12.7	34.7 ± 15	< .001*
**TL (°)**	3.6 ± 16.3	-22.1 ± 16.4	-18.6 ± 17.2	0.017*
**LL (°)**	3.8 ± 18.6	-70.3 ± 10.4	-64.1 ± 29.7	0.058
**LS (°)**	-2.0 ± 15.2	-27.7 ± 8.6	-29.5 ± 12.7	0.157
**CL (°)**	-22.3 ± 12.9	-7 ± 11.6	-15.3 ± 12.4	0.000*
**T1S (°)**	27.0 ± 12.1	14.4 ± 8.3	18.8 ± 10	< .001*
**C27 SVA (mm)**	16.1 ± 16.3	8.9 ± 10.5	9.9 ± 10.8	0.322
**T1S-CL**	4.7 ± 13.6	7.5 ± 8.7	3.5 ± 12	< .001*
**PI-LL**	59.9 ± 20.4	-14.2 ± 10.6	-8 ± 31.5	0.058
**SVA correction (Po-Pre)**	-	-201.1 ± 82.7	-	-
**TK correction (Po-Pre)**	-	23.1 ± 10.8	-	-
**LL correction (Po-Pre)**	-	-74.1 ± 19.4	-	-
**CL change (Po-Pre)**	-	15.3 ± 12.6	-	-
**T1S change (Po-Pre)**	-	-12.5 ± 10.7	-	-
**C2-7 SVA change (Po-Pre)**	-	-7.3 ± 14.3	-	-

Data are presented as mean ± standard deviation, or number.

Pre, preoperative; Po, postoperative (2months after surgery); Last F/U, last follow-up (2 years after surgery); PI, pelvic incidence; SS, sacral slope; PT, pelvic tilt; C7SVA, C7 plumb line sagittal vertical axis; TK, thoracic kyphosis; TL, thoracolumbar junctional angle; LL, lumbar lordosis; LS, lumbosacral junctional angle; CL, cervical lordosis; T1S, T1 slope.

* Statistically significant. (p-value < .05)

C7SVA was improved from +188.1 mm before surgery to -13 mm after surgery. Sagittal balance was maintained until the last follow-up with a C7SVA of +6.7 mm. TK was +2.6° before surgery, +25.7° after surgery, and +34.7° at the last follow-up. LL was +3.8° before surgery, -70.3° after surgery, and -64.1° at the last follow-up. The surgery corrected C7SVA to 201.1mm, TK to 23.1°, and LL to 74.1°.

The mean Pl was 56.1°. As LL was restored and PT maintained at the target value of < 20° for satisfactory alignment as defined by Schwab et al. [24], SS had increased after surgery and at the last follow-up.

CL was hyperlordotic at -22.3° before surgery and was reduced by a mean of 15.3° to -7° after surgery. However, Last CL had increased to -15.3°. T1S was reduced from 27° before surgery to 14.4° after surgery and had increased to 18.8° at the last follow-up. C27SVA was measured at 16.1 mm before surgery, 8.9 mm after surgery, and 9.9 mm at the last follow-up.

### Correlation and multilinear regression analysis of Post cervical parameters (Tables 3 and 4)

**Table 3:** Correlations between postoperative cervical lordosis, T1 slope, and other radiographic parameters.

	Pre TK	Pre LL	Po CSVA	Po T1S	Po C7SVA	SVA cor	Po TK	LL cor
**Po CL**	-0.145	0.334 **	-0.018	-0.657 **	-0.226 *	-0.239 *	-0.154	-0.314 **
**Po T1S**	0.371 **	-0.267 *	0.456 **		0.281 *	0.184	0.299 **	0.223 *

Pre, preoperative; Po, postoperative (2 months after surgery); CL, cervical lordosis; T1S, T1 slope; TK, thoracic kyphosis; LL, lumbar lordosis; CSVA, C27 sagittal vertical axis; C7SVA, C7 plumb line sagittal vertical axis; SVA, sagittal vertical axis; cor, correction.

* Significant correlations was established at the .05 level.

** Significant correlations was established at the .01 level.

**Table 4:** Multilinear regression analysis.

Variables	Factors	B	Significance	VIF
**Post CL**^**1**^^**)**^	Constant	-2.317	0.619	
	Post T1S	-0.863	0.000	1.052
	LL correction	-0.105	0.045	1.052
**Post T1S**^**2**^^**)**^	Constant	17.563	0.000	
	Post SVA	0.304	0.000	1.117
	Pre TK	0.138	0.033	1.133
	LL correction	0.083	0.049	1.017
**Last CL**^**3**^^**)**^	Constant	-5.051	0.065	
	Last T1S	-0.544	0.000	1.000
**Last T1S**^**4**^^**)**^	Constant	9.326	0.001	
	Last TK	0.273	0.000	1.000
**Last TK**^**5**^^**)**^	Constant	26.116	0.000	
	Post PI-LL	-0.596	0.000	1.000

Pre, preoperative; Post, postoperative (2 months after surgery); Last, last follow-up (2 years after surgery); CL, cervical lordosis; T1S, T1 slope; LL, lumbar lordosis; SVA, sagittal vertical axis; TK, thoracic kyphosis; PI, pelvic incidence.

1) r = .679, Durbin-Watson 2.2;

2) r = .549, Durbin-Watson 1.971;

3) r = .439, Durbin-Watson 2.072;

4) r = .410, Durbin-Watson 2.105;

5) r = .418, Durbin-Watson 1.581.

Post CL correlated with Pre LL, Post T1S, Post SVA, SVA correction and LL correction (p < .05). Post T1S correlated with Pre TK, Pre LL, Post CL, Post C27SVA, Post C7SVA, Post TK and LL correction (p < .05).

Multilinear regression analysis of these correlation factors allowed identification of the influencing factors significantly related to the Post CL (r = .679) and Post T1S (r = .549). After confirming the significance of each path, it was confirmed that the effects of Post T1S and LL correction were significantly associated with the Post CL (p < .05). The unstandardized beta (ß) coefficients of Post TIS (ß = -.863) and LL correction (ß = -.105) were negative, and it was found that the smaller the Post TIS and LL correction values, the larger the value of Post CL. The effects of Post SVA (ß = .304), Pre TK and LL correction were significantly associated with the Post T1S (p < .05). The unstandardized ß coefficients of Post SVA (ß = .304), Pre TK (ß = .138) and LL correction (ß = .083) were positive, and it was found that the larger Post SVA, Pre TK and LL correction values, the larger the value of Post T1S.

### Correlation and multilinear regression analysis of Last cervical parameters (Tables 4 and 5)

**Table 5:** Correlations between last follow-up cervical lordosis, T1 slope, thoracic kyphosis and other radiographic parameters.

	Last T1S	Po TK	Po LL	Po PI-LL	Po PT	Last TK	Last PT
**Last CL**	-0.439 **	-0.182	0.167	0.139	-0.098	-0.287 *	0.012
**Last T1S**		0.337 **	-0.258 *	-0.207	-0.073	0.410 **	-0.065
**Last TK**	0.410 **	0.774 **	-0.352 **	-0.418 **	-0.252 *		-0.245 *

Po, postoperative (2 months after surgery); Last, last follow-up (2 years after surgery); CL, cervical lordosis; T1S, T1 slope; TK, thoracic kyphosis; LL, lumbar lordosis; PI, pelvic incidence; PT, pelvic tilt.

* Significant correlations was established at the .05 level.

** Significant correlations was established at the .01 level.

Last CL correlated with Last T1S and Last TK (p < .05). Last T1S correlated with Post TK, Post LL, Last CL and Last TK (p < .05). Last TK correlated with Post TK, Post LL, Post PI-LL, Last PT and Last T1S (p < .05).

Multilinear regression analysis of these correlation factors allowed identification of the influencing factors significantly related to the Last CL (r = .439), Last T1S (r = .410) and Last TK (r = .418). After confirming the significance of each path, it was confirmed that the effect of Last T1S was significantly associated with the Last CL (p < .05). The unstandardized ß coefficient of Last T1S (ß = -.544) was negative, and it was found that the smaller the value of Last T1S, the larger the value of Last CL. The effect of Last TK was significantly associated with the Last T1S (p < .05). The unstandardized ß coefficient of Last TK (ß = .273) was positive, and it was found that the larger the value of Last TK, the larger the value of Last T1S. The effect of Post PI-LL was significantly associated with the Last TK (p < .05). The unstandardized ß coefficient Post PI-LL (ß = -.596) was negative, and it was found that the smaller the value of Post PI-LL, the larger the value of Last TK.

## Discussion

Dubousset described the ’Cone of Economy’ to explain the concept of optimal posture and standing balance [25], especially, sagittal balance which is an important factor allowing maintenance of stable posture using minimal energy [26, 27]. Therefore, restoration of sagittal balance is a key factor for the surgical management of ASD patients and the ‘chain of correlation’ extending from pelvic alignment to LL, TK, and CL has been increasingly recognized as an essential factor involved in maintaining sagittal balance [8, 9].

### Sagittal malalignment and compensation mechanism

Various compensatory mechanisms compensate for poor sagittal alignment [28, 29]. Since pelvic retroversion is caused by extensor muscles of the hip joint and lumbar spine, elderly patients with extensor weakness cannot compensate for sagittal imbalance due to the inability to rotate the pelvis backward. The next step in maintaining sagittal balance is compensation in the lower extremity which restores the sagittal imbalance through the extension of the hip joint and flexion of the knee [30–33]. Furthermore, cervical hyperlordosis may occur to maintain horizontal gaze [8]. In our study, patients showed severe sagittal imbalance with a mean C7SVA of 188.1 mm and a kyphotic LL of 3.8° (Pre Pl-LL: 59.9) before surgery. In compensating for the sagittal imbalance, patients exhibited hyperlordotic changes of CL (-23.3°) with SS reduction to 21.4°, PT increase to 34.6°, and TK reduction to 2.6°.

### Reciprocal change and cervical spine

Surgical treatment of sagittal malalignment improves the health-related quality of life of patients and prevents further decompensation [1, 2, 34]. However, changes in unfused segments that occur as the patient adapts to the standing posture after reconstruction surgery pose another challenge for spine surgeons. Many studies have discussed these reciprocal changes occurring in the pelvis, thoracic and lumbar regions [10–13]. Reciprocal changes can also occur in the cervical spine following deformity correction; however, the mechanism by which these changes occur is not yet clear.

The cervical spine is a complex structure which supports the head and shows a relatively wide range of motion compared with other spine segments [8, 35], and this dynamic nature of the cervical spine can lead to cervical hyperlordosis when sagittal malalignment occurs. Depending on the extent of surgical sagittal correction, cervical hyperlordosis may be reduced, and in severe cases, kyphosis can result [14, 15]. Studies conducted to date have provided limited insights due to differences in the age of patients examined, etiologies, fusion levels, and surgical techniques used. On the other hand, we examined patients with a single etiology, having the same fusion level from T10 to S1, and in whom lumbosacral junctional stability was increased by sacropelvic fixation and L5-S1 interbody fusion. No significant change in LL was observed after surgery and at the last follow-up. PT and SS also showed favorable outcomes (Table 2, stabilization of the pelvic parameters through optimal LL correction). Thus, we were able to more reliably examine the changes in cervical lordosis after surgery and at the last follow-up, along with factors that affect these changes.

### Single etiology of lumbar degenerative kyphosis and cervical reciprocal change

LDK is a type of ASD characterized by severe sagittal malalignment and is relatively common in Asian countries. Yagi *et al*. [16] recently redefined LDK, which is kyphosis of the lumbar region caused by degenerative changes in the spine, muscle, and ligamentous complex, as ‘dropped body syndrome’. LDK clearly differs from idiopathic scoliosis and iatrogenic fixed sagittal imbalance in terms of patient population, etiology, and biomechanics. A study on spinopelvic parameters of LDK by Lee et al. [36] reported that patients with LDK had a high PI of >50°, as defined by Roussouly and Pinheiro-Franco [37], and that SS and LL in LDK patients were relatively low, while PT had relatively increased. In our study, patients also had a high PI with a mean value of 56.1°. Therefore, the patients underwent lordosis correction by a mean LL of 74.1° in consideration of their high initial PI. Radiographic improvements in C7SVA, PT, and TK were observed following lumbar lordosis correction and at the last follow-up. Furthermore, a drastic cervical kyphotic change of 15.3° was also observed.

**Fig 1:**
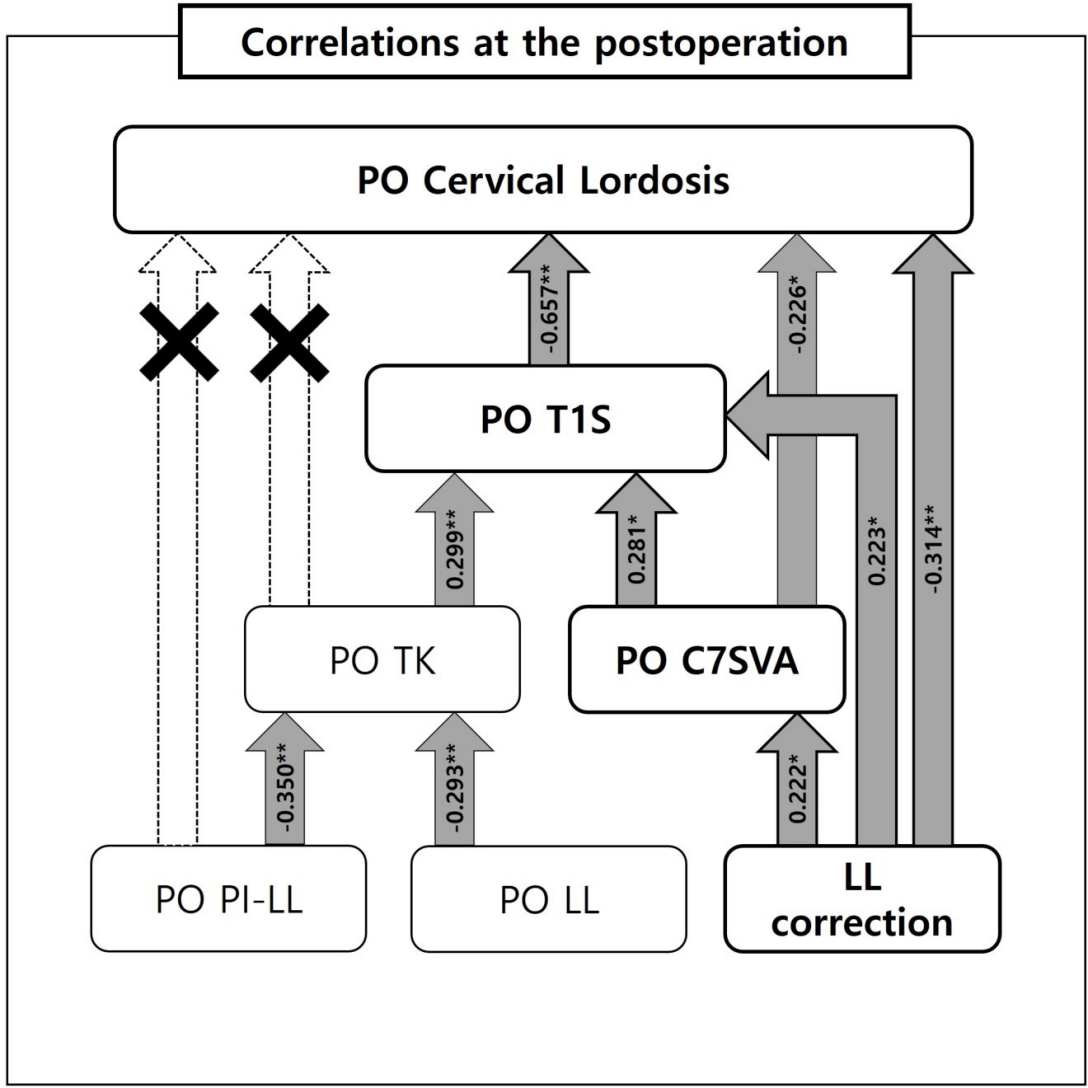
Correlations between Post cervical parameters and the various spinopelvic parameters. Post CL and Post T1S were more significantly affected by the amount of LL correction than by the changes in Post LL, Post TK and Post PI-LL.

Through a correlation test and multilinear regression analysis (Tables 3 and 4), we found that Post CL and T1S were more significantly affected by the amount of LL correction than by the changes in Post LL, Post TK and Post PI-LL (Fig 1). Thus, we concluded that patients in our study underwent a relatively large LL correction due to high PI, leading to a drastic reduction in Post C7SVA and subsequently Post T1S reduction. The reduction in Post T1S consequently led to the kyphotic change in CL. Some studies suggest that persistent kyphotic changes in CL can lead to cervical deformities over time [38]. However, most of these studies included patients whose UIV levels were in the upper thoracic region, whereas the patients in our study had a UIV level of T10. When the UIV level is upper thoracic, postoperative cervical kyphotic changes may be more significantly affected by the surgical technique or rod contouring rather than the reciprocal change in the unfused segment [11]. Unlike in previous studies, patients’ CL was restored to -15.3° at the last follow-up, and cervical kyphosis did not worsen in our study.

**Fig 2:**
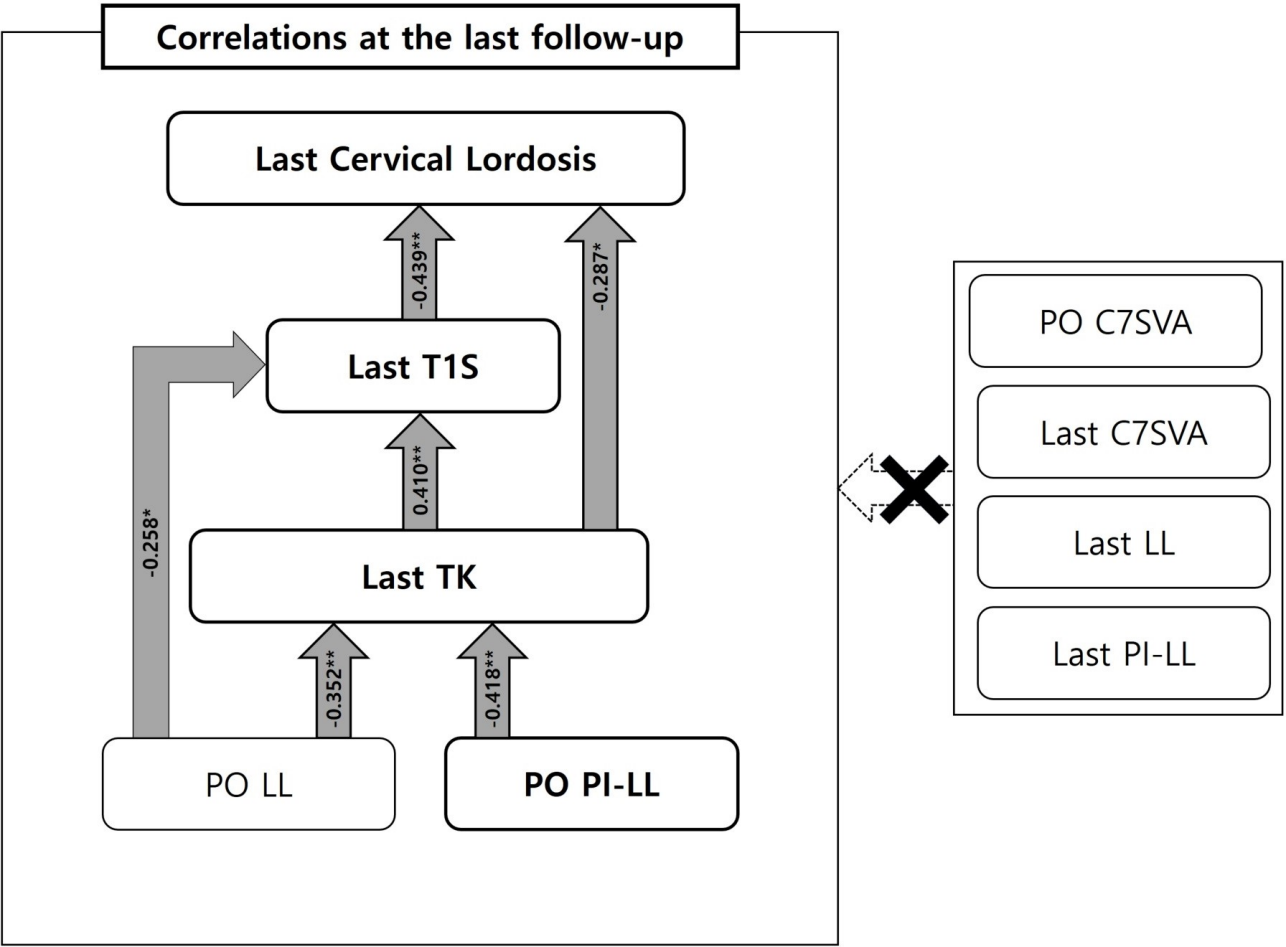
Correlations between Last cervical parameters and the various spinopelvic parameters. Last CL was affected by Last T1S, and Last T1S was affected by Last TK, but both parameters were not significantly associated with Post and Last C7SVA, Last LL and Last PI-LL. Additionally, there was a significant association between Last TK and Post PI-LL.

**Fig 3:**
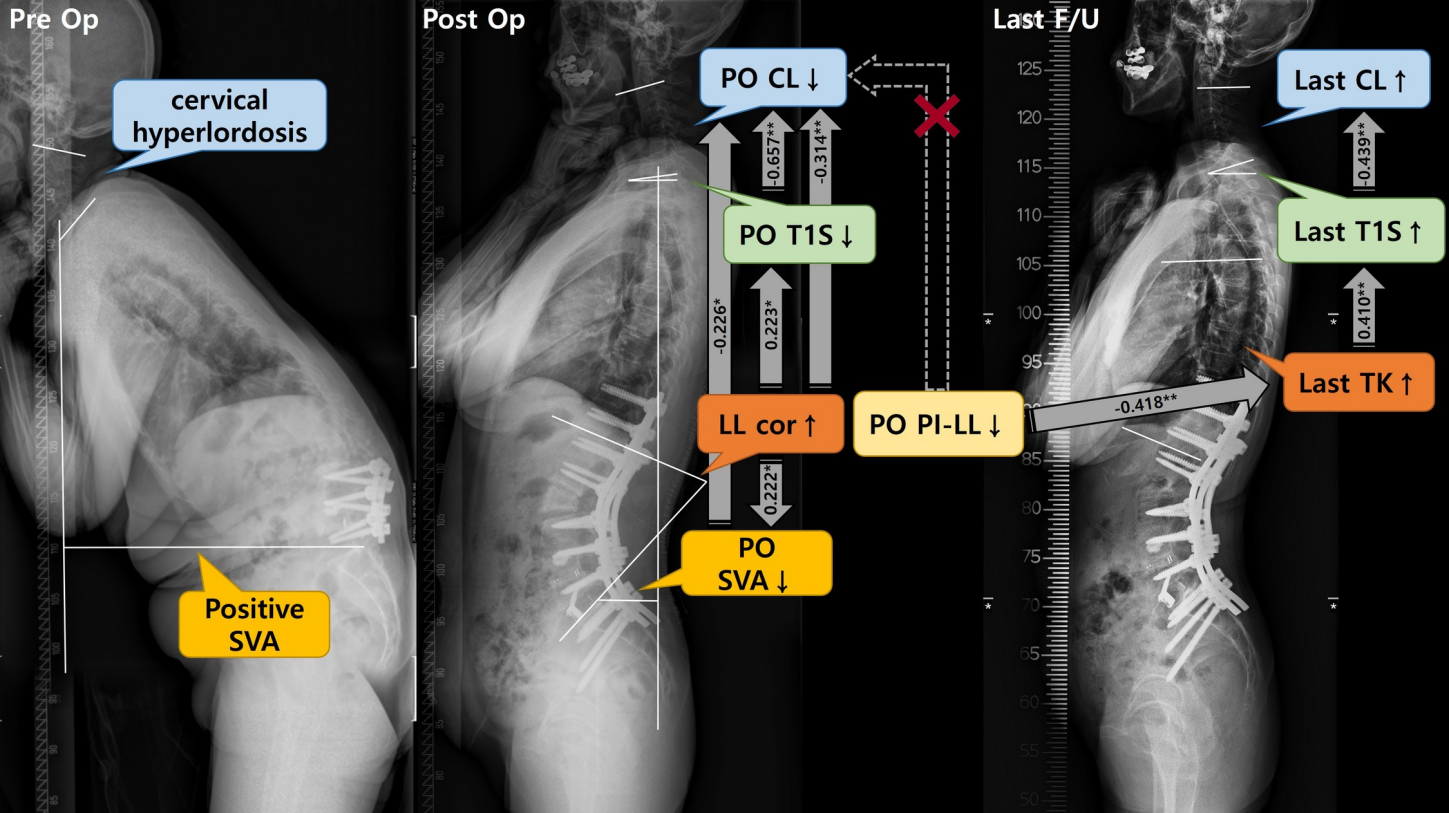
Case. A 62-year-old female with degenerative sagittal imbalance and cervical hyperlordosis (C7SVA + 293mm, TK -4°, LL 21°, PT 29°, PI 51°, CL -30°, T1S 45°) underwent PSO on L2, PLIF on L4-5, ALIF on L5-S1, and posterior spinal fusion from T10 to S1 with sacropelvic fixation and accessory rod fixation. Optimal sagittal alignment was obtained after correction (C7SVA -48mm, TK 12°, LL -65°, PT 5°), but cervical kyphosis occurred (CL +16°, T1S 10°). A relatively large amount of LL correction due to the high PI led to a drastic reduction in Post C7SVA and subsequent reduction of Post T1S and CL. Radiograph at 2 years postoperatively showed a well-maintained optimal sagittal alignment with restoration of cervical lordosis (C7SVA -24mm, TK 27°, LL -66°, PT 5°, CL -5°, T1S 15°). TK increased to achieve spinopelvic harmony resulting from the postoperative difference in LL relative to PI. This modification in TK led to gradual optimal changes in T1S and CL over time.

We performed a correlation test and multilinear regression analysis on Last CL and Last T1S to investigate factors which affect the restoration of CL over time in patients whose CL showed drastic kyphotic change after surgery (Tables 4 and 5). We found that Last CL was affected by Last T1S, and Last T1S was affected by Last TK. However, both parameters were not significantly associated with Post and Last C7SVA, Last LL and Last PI-LL (Fig 2). Based on these results, we found that TK increased over time after surgery, and that the increase in TK affected the increase in T1S, thereby restoring CL. We conducted an additional analysis to investigate factors affecting Last TK (Tables 4 and 5) and found a significant association between Last TK and Post PI-LL. Consistent with the findings of Jang et al. [39], we found a reciprocal relationship between LL and TK, and deduced that TK increased to achieve spinopelvic harmony resulting from the postoperative difference in LL relative to PI. Therefore, this modification in TK led to gradual optimal changes in T1S and CL over time (Fig 3).

A number of studies have reported that changes in TK after deformity correction have a negative impact on sagittal alignment including PJK [10]. One study described postoperative changes in TK as having an ‘unpredictable nature’ [40]. While there are conflicting opinions in the literature regarding the changes in TK after deformity correction, the patients in our study exhibited spinopelvic harmony in regions including the cervical area. Our study patients maintained optimal sagittal balance with a mean C7SVA of 6.7 mm and optimal PT of 14.5° until the last follow-up. Thus, the gradual increase in TK in patients who showed spinopelvic harmony after deformity correction is considered a positive change in the maintenance of optimal balance until the last follow-up.

### Limitations

There were several limitations to this study. First, because this study was a retrospective study, not all variables involved in the effects of kyphotic change of CL followed by lordosis restoration on the cervical spine could be considered or evaluated. Second, because our study patients were operated on by a single surgeon at a single institution, the results may have limited implications. Third, the improvement in quality of life following deformity correction may have hindered the diagnosis of symptoms and disabilities caused by cervical disorders. In our study, four patients (1 patient with C1-2 instability, 2 with herniated cervical intervertebral disc and 1 with C4-7 spinal stenosis with ossification of posterior longitudinal ligament) had a cervical disorder, and they all showed improvements after conservative treatment. It appears that the incidence of cervical deformities among these patients was relatively low because the patients were not included in the cervical deformity classification criteria developed by Passias et al. [38] which specifies TS-CL >20°, C27SVA >40 mm, or C2–C7 kyphosis >10° before and after surgery and at the last follow-up. Forth, our results showed a relatively high PI (>50°), indicating that an LL correction of >50° is feasible. However, in the case of the patients with low PI of type 1 and 2 as in the study by Roussouly and Pinheiro-Franco [37] or relatively small LL correction due to the mild sagittal imbalance, the results may be different. For example, the results of the study by Neuman et al. [41] on the reciprocal changes of the cervical alignment after thoracolumbar arthrodesis in 171 ASD patients with mild sagittal imbalance (C7SVA 60 mm) and mild compensatory changes in LL (-41°), TK (32°) and PT (23°) before surgery, there was decrease in C2-7 lordosis from -9.3° (baseline) to -7.9° at 2 years. However, comparing before and after surgery, and at the last follow-up, CL also decreased from -9.3° before surgery to -6° at 6 weeks after surgery, but increased to 7.9° at 2 years after surgery. This result was consistent with our results showing that CL had decreased soon after ASD surgery but had increased gradually over time.

## Conclusion

The results of this study demonstrated that the postoperative kyphotic CL changes in ASD patients with cervical hyperlordosis preoperatively were affected by drastic LL correction and SVA restoration, and that these changes were not permanent. Furthermore, to achieve spinopelvic harmony proportional to the difference in LL relative to PI, TK was modified over time to increase T1S and CL. The results of this study can be used as a guideline for spine surgeons who plan and make decisions regarding spine reconstruction surgery for patients with ASD.

## Supporting information

S1 File Raw data source for influencing radiographic factors related to the cervical parameters (data are provided as a MS-Excel data sheet file). (XLSX)
